# Lessons from the Minab tragedy: protecting education in the age of AI-assisted warfare

**DOI:** 10.3389/frai.2026.1914374

**Published:** 2026-09-18

**Authors:** Ali Meghdari

**Affiliations:** 1 Islamic Azad University-Fereshtegaan International Branch, Tehran, Iran; 2 Sharif University of Technology, Tehran, Iran

**Keywords:** AI-assisted warfare, international laws, Minab, protecting education, Shajareh Tayyebeh Elementary School

“The destruction of the Shajareh Tayyebeh Elementary School in Minab, Iran, on February 28, 2026, should force the international community to confront a painful truth: even the most technologically advanced militaries remain capable of catastrophic errors when civilians are insufficiently protected. This article aims to elaborate on lessons learned from Minab and similar tragedies, hoping that although the scientific community cannot by itself prevent wars, it can influence how evidence is gathered; technologies are developed; and accountability is pursued.”

On February 28, 2026, what began as an ordinary school day at Shajareh Tayyebeh Elementary School ended in unimaginable tragedy when United States and Israeli missiles “according to multiple public reports and ongoing investigations,” struck the school grounds, causing the collapse of large sections of the building and trapping hundreds of children, teachers, and staff beneath the rubble. According to multiple independent investigations, at least 168 people were killed, many of them young students between the ages of 7 and 12 (Meghdari, 2026).^1^^−^^7^

The school, known for serving children from the local community, was filled with students attending classes when the attack occurred. Witnesses described scenes of panic as explosions shook the area. Teachers reportedly rushed to protect their pupils, guiding them toward safer parts of the building and attempting to contact parents. Moments later, additional strikes reportedly hit the vicinity, turning the school into a scene of destruction and chaos. Rescue workers, emergency personnel, and local residents immediately began searching through the debris. Images and videos from the aftermath showed shattered classrooms, torn schoolbooks, children's backpacks, and desperate efforts to locate survivors. Families gathered outside the ruins, hoping for news of their children, while hospitals struggled to cope with the large number of casualties and injuries. Due to the distasteful and offensive real images of this catastrophe, an AI generated picture of Shajareh Tayyebeh Elementary School before and after bombardment is shown in Figure 1 to virtually describe the level of crime and destruction.

**Figure 1:**
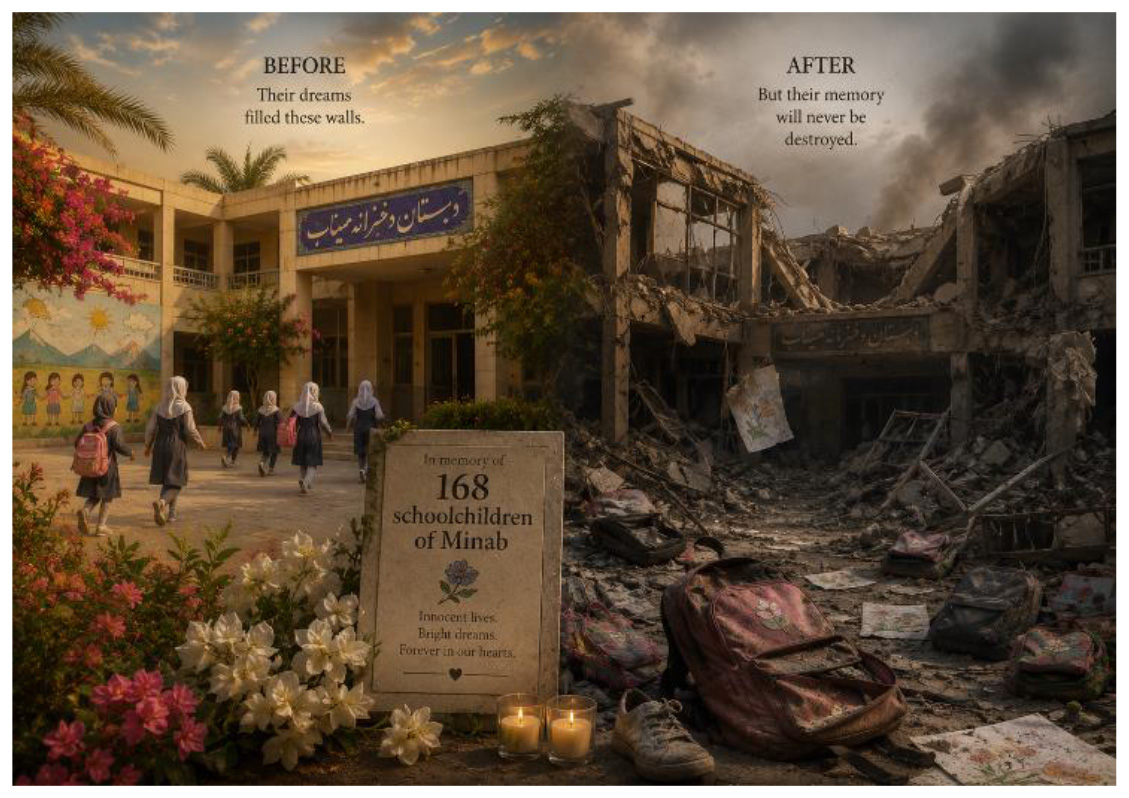
AI generated by ChatGPT.

The tragedy sparked widespread grief across Iran and drew international attention. Human-rights organizations condemned the loss of civilian life and called for independent investigations into the circumstances of the attack. Several groups argued that schools are protected civilian sites under international humanitarian law and that all parties to a conflict must take every possible precaution to prevent harm to children. For the people of Minab, the attack left scars that extend far beyond the physical destruction of a school building. Classrooms that once echoed with laughter and learning became symbols of loss. Parents mourned children whose futures were cut short, classmates grieved friends they would never see again, and an entire community struggled to come to terms with the scale of the devastation.

The Minab school tragedy stands as a painful reminder of the human cost of armed conflict. Regardless of political disputes and military objectives, the deaths of so many children underscore the profound consequences that war can have on civilians. As investigations and debates continue, the memory of the students, teachers, and families affected by the attack remains a powerful call for accountability, protection of civilians, and the pursuit of peace.

The reported destruction of a school in Minab, Iran, and the deaths of large numbers of children have drawn international attention to the vulnerability of educational institutions in conflict zones. While investigations and public accounts continue to examine the circumstances of the incident and questions of responsibility, the event highlights a broader and well-documented concern: the devastating impact of armed conflict on children and their access to education. Schools serve not only as centers of learning but also as places of safety, social development, and community cohesion. When educational facilities are damaged or destroyed during military operations, the consequences extend far beyond the immediate casualties. Survivors often experience long-term psychological trauma, interrupted education, displacement, and reduced access to essential social services. International humanitarian law provides special protections for civilians and civilian infrastructure, including schools. Numerous international organizations have documented the increasing risks faced by children in conflict-affected regions and have called for stronger measures to prevent attacks on educational facilities. Such incidents raise urgent questions regarding civilian protection, accountability, and compliance with established legal norms (Meghdari, 2026).

The reported Minab tragedy certainly illustrates how a single event can affect an entire generation (see text footnote 6 and 7). Families, friends, educators, healthcare workers, and local communities bear the burden of recovery long after media attention fades. Figure 2 is a painting by an Iranian artists drawn in memory of the innocent children who have lost their life in this tragedy. Beyond the immediate loss of life, the destruction of educational environments can undermine social stability and hinder long-term development. For researchers, policymakers, and humanitarian organizations, incidents involving schools underscore the importance of systematic data collection, independent investigation, and evidence-based policy responses. Understanding the factors that place children at risk during armed conflict is vital for developing effective prevention strategies and strengthening international mechanisms for civilian protection. Regardless of political perspectives, the protection of children and educational institutions remains a universal humanitarian imperative. Continued research, transparent investigations, and global cooperation are necessary to reduce the chance of similar tragedies and ensure that schools remain places of learning rather than casualties of war.

**Figure 2:**
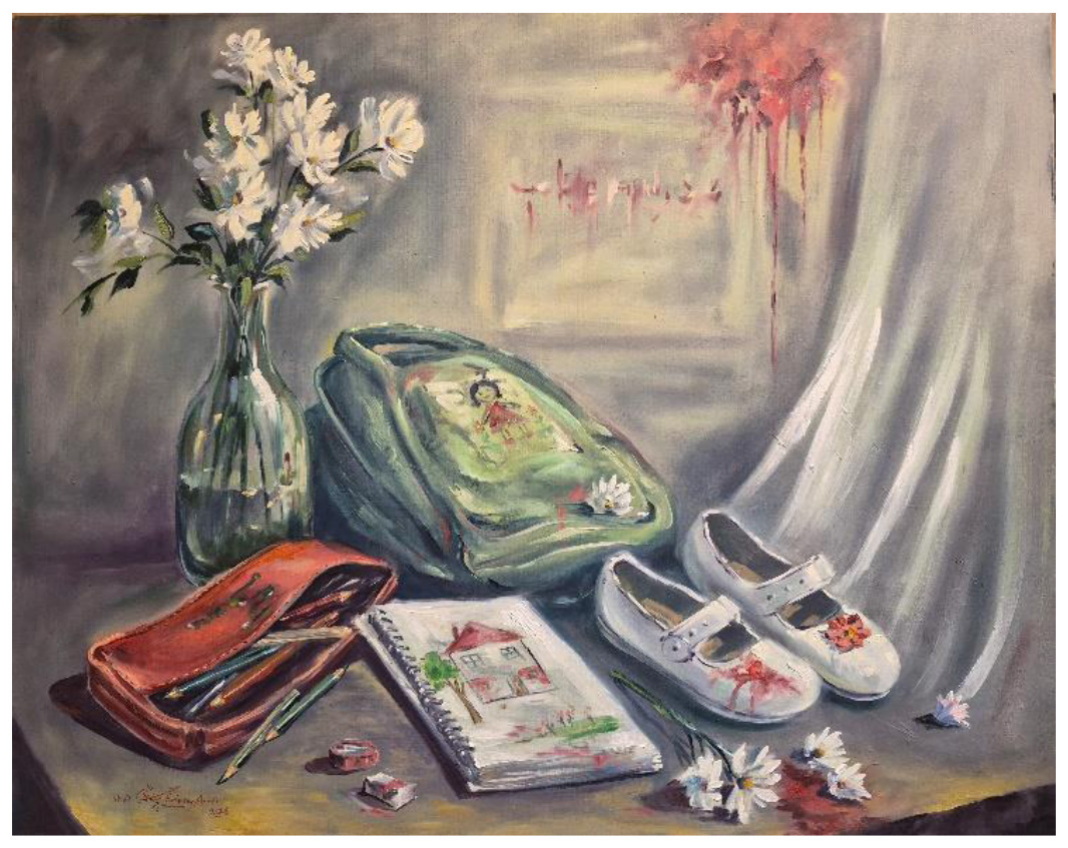
Painted by Farhang Atefi (Image in this figure is a painting by Iranian Artist Dr. Farhang Atefi, Fereshtegaan International University, Tehran, Iran, June 13, 2026). Published with permission.

The tragedy is not simply a story about one school. It is a warning about the growing gap between the sophistication of modern weapons and the reliability of the information systems that guide them. Military planners increasingly rely on vast streams of intelligence data, satellite imagery, automated analysis, and algorithm-assisted decision-making. These technologies promise greater precision and fewer civilian casualties. Nevertheless, the events in Minab demonstrate that precision is meaningful only when the underlying information is accurate and continuously verified. A missile can strike within meters of its intended coordinates and still produce a humanitarian disaster if the coordinates themselves are wrong. Reports surrounding the Minab strike suggest that the school stood near facilities associated with Iran's Revolutionary Guards and that historical information about the area may have influenced targeting assessments. Whether future investigations ultimately attribute the disaster to outdated intelligence, procedural failures, technological shortcomings or human error, the broader lesson remains the same: civilian protection cannot be delegated to assumptions embedded in databases.

This challenge extends beyond the Middle East. Around the world, governments are investing heavily in artificial intelligence, autonomous systems and data-driven military planning. Advocates often emphasize speed, efficiency and operational advantage. Far less attention is devoted to the consequences of incorrect data, degraded intelligence or flawed classification systems. When errors occur, they can be amplified by the very technologies designed to improve decision-making. The deaths of children in Minab should therefore be viewed not only as a humanitarian tragedy but also as a policy failure. International law already requires belligerents to distinguish between military objectives and civilian objects and to take all feasible precautions to minimize civilian harm. The question is whether existing accountability mechanisms are keeping pace with rapidly evolving military technologies.

The growing integration of artificial intelligence into military decision-support systems raises an additional challenge for International Humanitarian Law. The legal principle of proportionality requires commanders to determine whether the anticipated incidental civilian harm would be excessive in relation to the concrete and direct military advantage expected from an attack. This assessment is inherently qualitative, context-dependent, and ultimately a matter of human legal judgment rather than algorithmic optimization. AI systems may assist by processing large volumes of intelligence or identifying potential military objectives, but they cannot independently evaluate the moral, legal, and contextual considerations that proportionality requires. Moreover, AI does not merely provide neutral information. By determining which information is highlighted, how confidence levels are presented, and how rapidly decisions are expected to be made, AI systems actively reshape the conditions under which human judgment is exercised. Automation bias, opaque model behavior, embedded biases in training data, and increasing operational tempo may unintentionally narrow the deliberative space required for careful legal assessment. Ensuring meaningful human control therefore requires more than retaining a human “in the loop”; it requires designing AI-supported decision processes that preserve independent legal judgment, transparency, and accountability.

Three reforms deserve urgent consideration. First, countries should adopt stricter independent review procedures for targets located near schools, hospitals, and other protected civilian sites. Second, military investigations involving large-scale civilian casualties should be transparent and subject to external scrutiny. Third, governments developing AI-assisted targeting systems should be required to disclose how such systems are validated, audited, and monitored for error.

The children who died in Minab will never benefit from these reforms. However, their deaths should compel policymakers, military leaders and researchers to examine whether current practices are adequate for an era in which a single targeting mistake can extinguish hundreds of futures in a matter of seconds. Scientific and technological progress is often celebrated for expanding human capability. The true measure of that progress, however, is whether it also expands human responsibility. Minab reminds us that the answer remains uncertain. The consequences of attacks on children extend far beyond the immediate death toll. Every classroom contains a reservoir of future human potential that cannot be measured at the moment of loss. Among the students killed in Minab may have been future scientists, physicians, engineers, teachers and innovators whose contributions could have benefited not only their own communities but humanity as a whole.

Scientific progress depends on the emergence of talent from diverse social, economic and geographical backgrounds (Meghdari, 2025). History repeatedly demonstrates that transformative discoveries often come from unexpected places. The next breakthrough in cancer treatment, renewable energy, climate adaptation, artificial intelligence or public health may originate from a child attending a school far from the world's major research centers. When conflict destroys educational opportunities or claims young lives, society loses not only individuals but also the knowledge, creativity and leadership they might have contributed. The effects are cumulative. Beyond those who perish, surviving classmates may experience trauma, displacement and interruptions to their education. Schools may close, teachers may leave, and families may become reluctant to send children to class. The resulting decline in educational attainment can persist for decades, reducing the number of students who ultimately enter higher education, research and innovation. In this way, a single attack can reverberate through an entire generation. For the global scientific community, the protection of children and educational institutions is therefore not solely a humanitarian concern. It is also an investment in humanity's future intellectual capacity. Every child denied the opportunity to learn represents a possibility lost; every school destroyed narrows the pipeline through which future discoveries emerge. The cost of such violence cannot be calculated only in lives lost today, but also in knowledge, solutions, and progress that the world may never realize.

Although the scientific community cannot by itself prevent wars, it can influence how evidence is gathered, how technologies are developed, and how accountability is pursued. For this purpose, several avenues may be proposed as follows:

**1. Establish an international scientific observatory for attacks on education**

The global research community could support an independent, internationally governed system that documents attacks on schools, universities, hospitals, and other civilian institutions. Using satellite imagery, geospatial analysis, open-source intelligence, and forensic methods, such a network could provide rapid, evidence-based assessments of incidents. Reliable documentation can reduce misinformation, preserve evidence, and support future accountability processes.

**2. Develop trustworthy AI assurance frameworks for military decision-support**

Scientists, engineers, and legal scholars should collaborate in developing internationally recognized AI assurance frameworks for military decision-support systems. These frameworks should include: auditable workflows documenting how AI contributes to operational decisions; system cards describing intended use, limitations, uncertainty, training data, and known biases; continuous monitoring for concept drift, performance degradation, and changing operational environments; independent verification that AI-assisted systems remain compatible with International Humanitarian Law throughout their operational life cycle rather than only during initial deployment.

The scientific community has historically shaped norms in areas such as nuclear weapons, biotechnology, and climate science. Similar leadership could help establish norms for AI-assisted warfare.

**3. Create a global “protected education infrastructure” database**

Researchers in geospatial science, remote sensing, and information systems could help maintain verified, continuously updated maps of schools, universities, and other protected civilian sites. If integrated into military planning systems worldwide, such databases could serve as an additional safeguard against accidental targeting.

**4. Expand research on civilian-harm mitigation**

Compared with investment in weapons development, relatively little research funding is devoted to understanding and preventing civilian casualties. Governments, foundations, and academic institutions could support multidisciplinary research involving: conflict studies, artificial intelligence, human rights law, data science, risk assessment, psychology, and trauma recovery. The goal would be to identify systematic ways to reduce harm before military operations occur.

**5. Strengthen scientific diplomacy**

Even during periods of geopolitical tension, scientific cooperation often survives when political dialogue breaks down. International scientific organizations such as the International Science Council and agencies within United Nations frameworks can help maintain channels of communication between countries in conflict. Although scientific diplomacy cannot prevent every war, it can create relationships and trust that reduce misunderstandings and encourage de-escalation.

**6. Protect education as a global public good**

The scientific community can advocate for schools and universities to be treated similarly to internationally protected cultural and humanitarian sites. This could include: enhanced monitoring, rapid international investigations after incidents, stronger legal protections, greater political consequences for violations.

**7. Support affected generations**

Prevention is crucial, but so is recovery. Scientists, universities, and funding agencies can provide scholarships, remote learning opportunities, research fellowships, and educational support for children affected by conflict. This helps ensure that the loss of educational opportunity does not become a second tragedy following the initial violence.

**8. Strengthen Article 36 weapons reviews for AI-enabled systems**

States developing AI-assisted targeting technologies should ensure that Article 36 reviews under Additional Protocol I extend beyond conventional weapons to encompass the entire AI-enabled decision-support process. Because machine-learning systems may evolve after deployment through changing data distributions and operational conditions, legal review should be an ongoing rather than one-time process. Scientists can contribute methodologies for continuous validation, uncertainty estimation, and independent auditing (Langeveldt and Pietersen, 2024; Boulanin, 2015).

**9. Support international accountability and protection of education**

Although existing international laws prohibit the targeting of civilian infrastructure, and places such as schools and hospitals, a major problem, particularly in recent years, is that these laws are being flouted openly by governments with little to no consequences. So, while it is important to develop regulations related to the design and use of AI, scientists, engineers, legal scholars, and politicians also need to make sure their governments follow the existing rules. In addition, scientific organizations should collaborate with international bodies investigating attacks on educational institutions by providing technical expertise in digital forensics, satellite imagery, geospatial analysis, and data verification.

In conclusion: The scientific community cannot determine where wars are fought, but it can influence how knowledge is used within them. Scientists have helped humanity split the atom, map the genome, and explore distant planets. The same ingenuity should be directed toward a more immediate challenge: ensuring that advances in technology reduce, rather than amplify, the risk to children caught in conflict. Protecting schools is not only a humanitarian obligation; it is a commitment to preserving the minds that will shape humanity's future.

## Footnotes

1
https://www.amnestyusa.org/blog/u-s-responsible-for-killing-over-100-children-in-iran-school-attack/

7
https://www.nbcnews.com/world/iran/know-strike-school-iran-death-toll-rises-rcna261266
